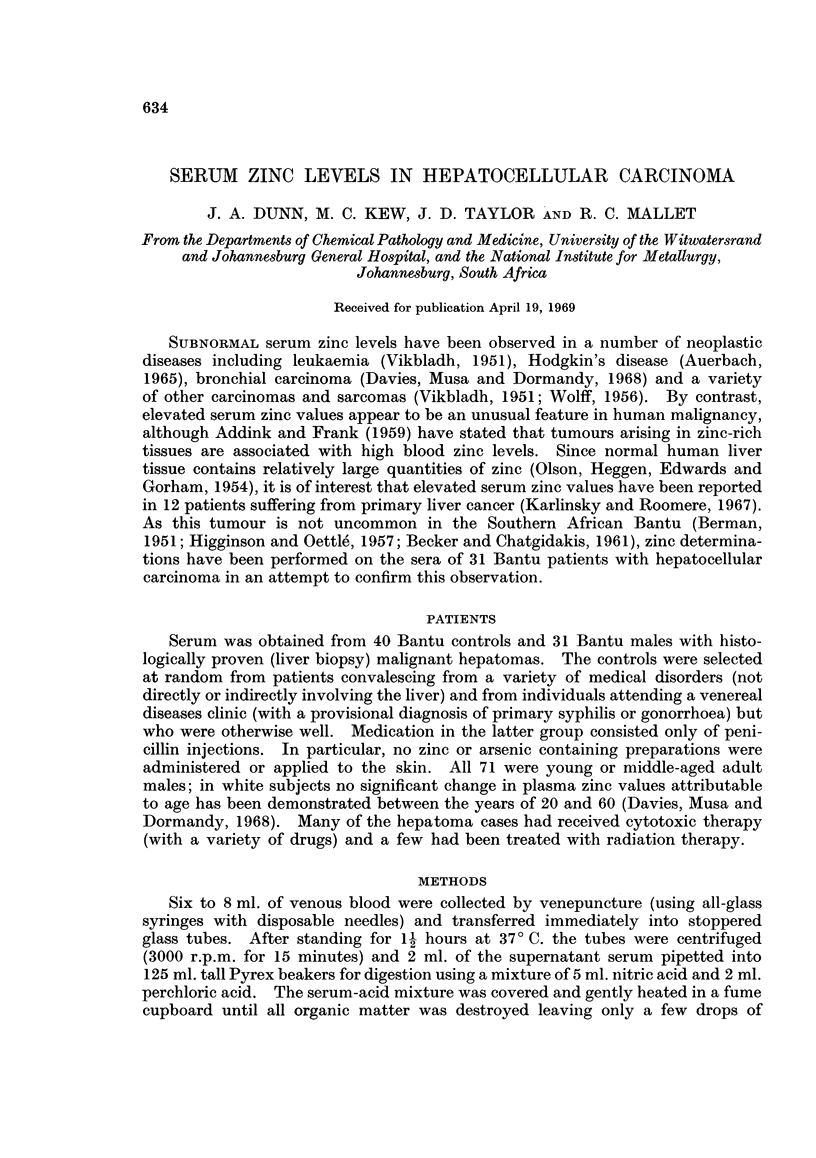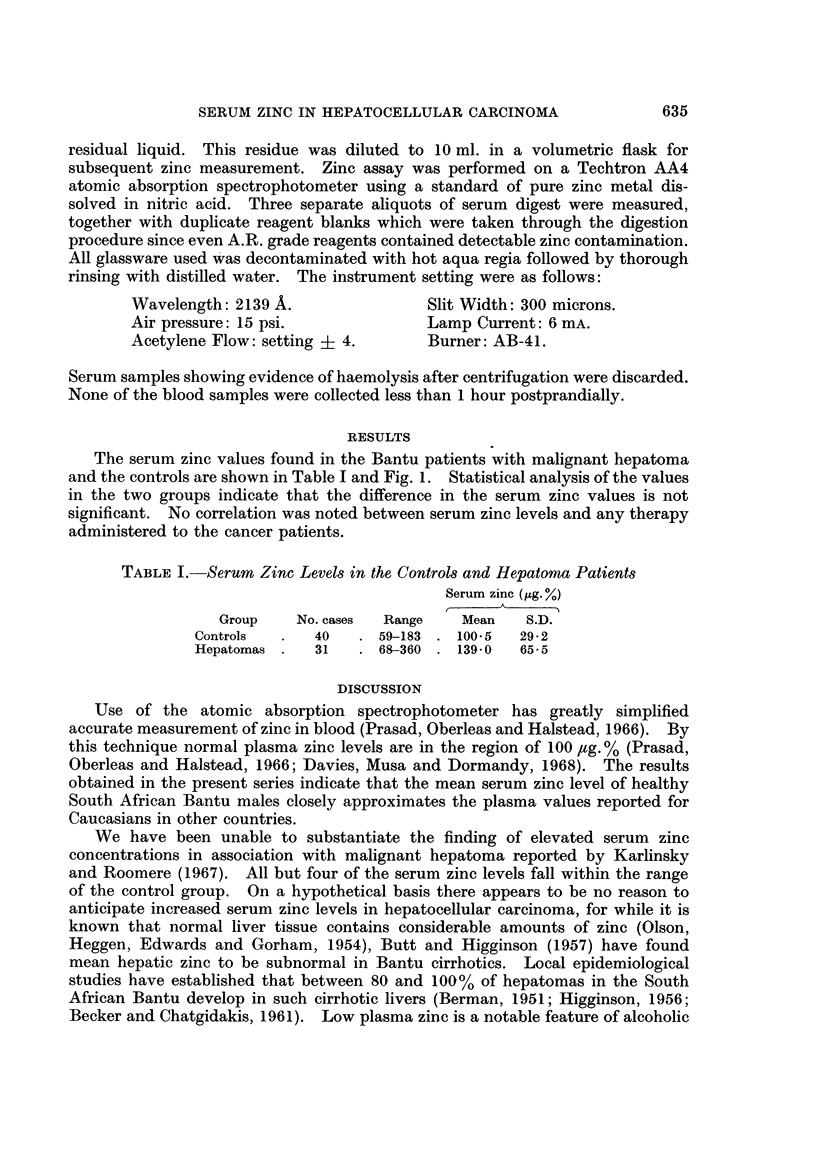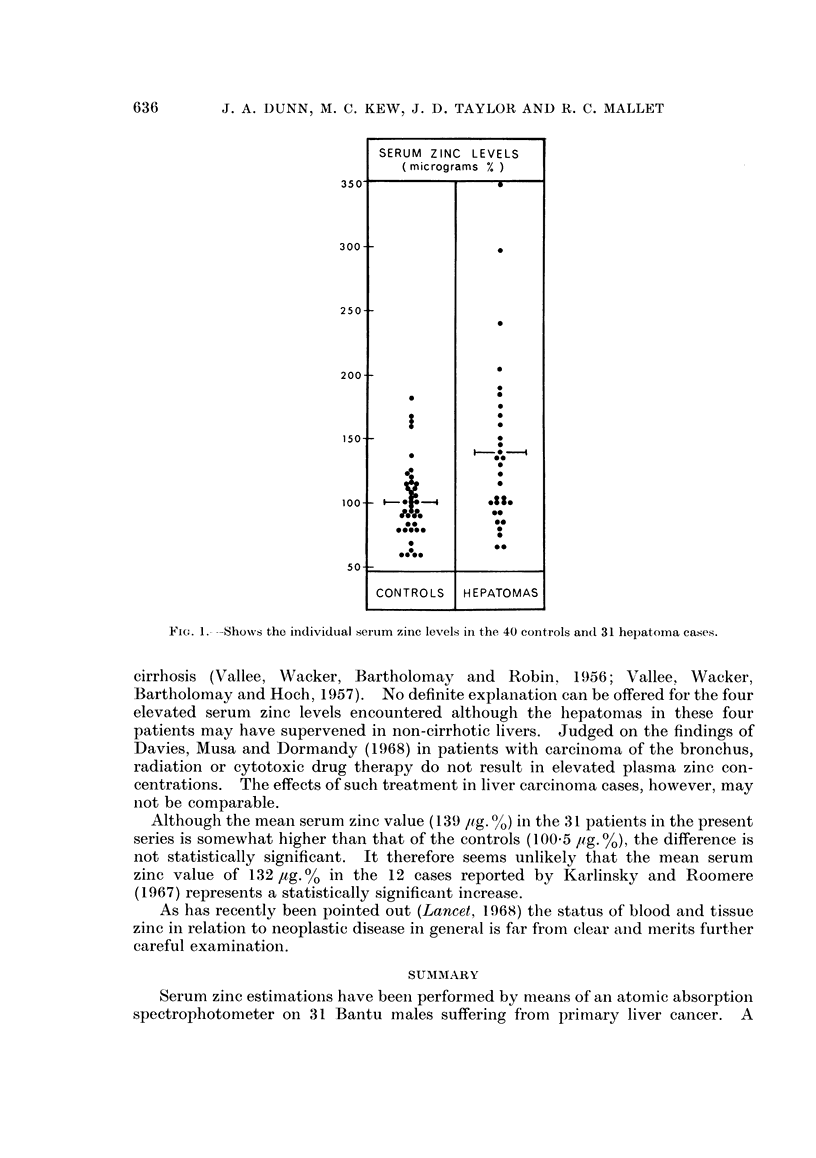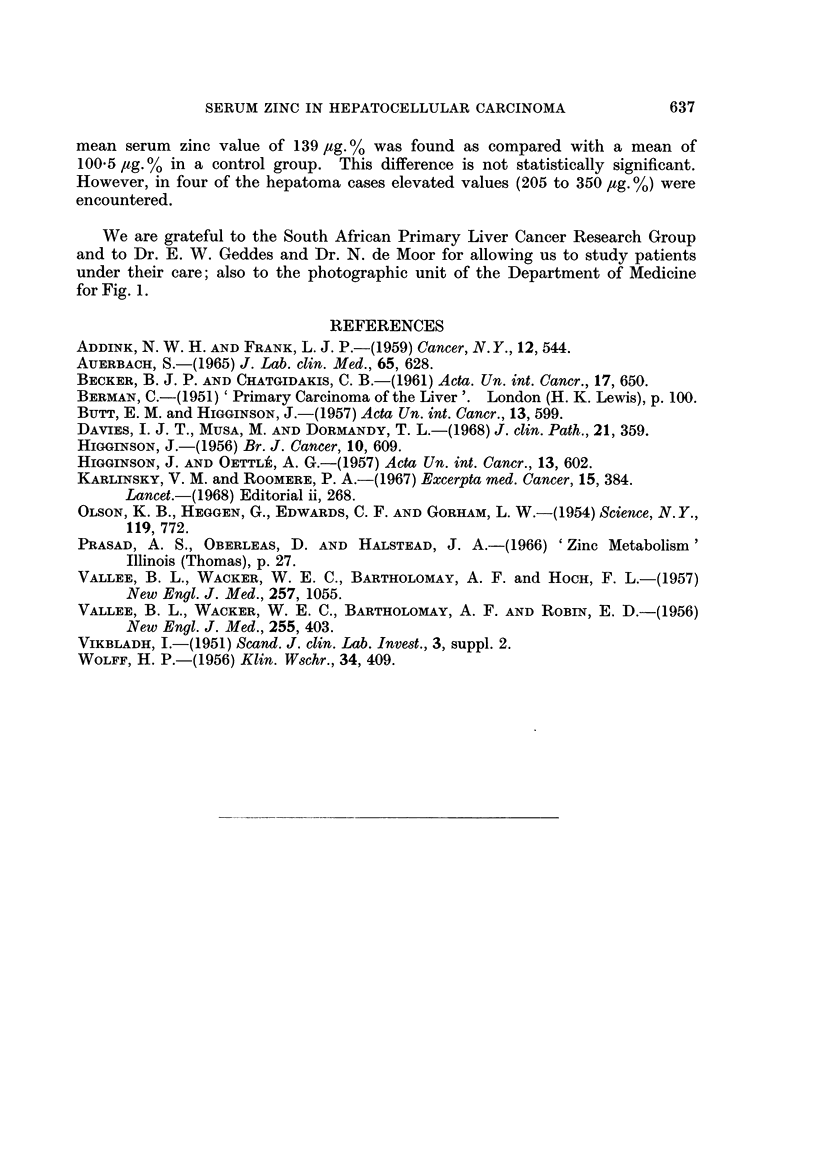# Serum zinc levels in hepatocellular carcinoma.

**DOI:** 10.1038/bjc.1969.78

**Published:** 1969-09

**Authors:** J. A. Dunn, M. C. Kew, J. D. Taylor, R. C. Mallet


### SERUM ZINC LEVELS IN HEPATOCELLULAR CARCINOMA

J. A. DUNN, M. C. KEW, J. D. TAYLOR AND R. C. MALLET

*From the Departments of Chemical Pathology and Medicine, University of the Witwatersrand and Johannesburg General Hospital, and the National Institute for Metallurgy, Johannesburg, South Africa*

Received for publication April 19, 1969

SUBNORMAL serum zinc levels have been observed in a number of neoplastic diseases including leukaemia (Vikbladh, 1951), Hodgkin's disease (Auerbach, 1965), bronchial carcinoma (Davies, Musa and Dormandy, 1968) and a variety of other carcinomas and sarcomas (Vikbladh, 1951; Wolff, 1956). By contrast, elevated serum zinc values appear to be an unusual feature in human malignancy, although Addink and Frank (1959) have stated that tumours arising in zinc-rich tissues are associated with high blood zinc levels. Since normal human liver tissue contains relatively large quantities of zinc (Olson, Heggen, Edwards and Gorham, 1954), it is of interest that elevated serum zinc values have been reported in 12 patients suffering from primary liver cancer (Karlinsky and Roomere, 1967). As this tumour is not uncommon in the Southern African Bantu (Berman, 1951; Higginson and Oettlé, 1957; Becker and Chatgidakis, 1961), zinc determinations have been performed on the sera of 31 Bantu patients with hepatocellular carcinoma in an attempt to confirm this observation.

### PATIENTS

Serum was obtained from 40 Bantu controls and 31 Bantu males with histologically proven (liver biopsy) malignant hepatomas. The controls were selected at random from patients convalescing from a variety of medical disorders (not directly or indirectly involving the liver) and from individuals attending a venereal diseases clinic (with a provisional diagnosis of primary syphilis or gonorrhoea) but who were otherwise well. Medication in the latter group consisted only of penicillin injections. In particular, no zinc or arsenic containing preparations were administered or applied to the skin. All 71 were young or middle-aged adult males; in white subjects no significant change in plasma zinc values attributable to age has been demonstrated between the years of 20 and 60 (Davies, Musa and Dormandy, 1968). Many of the hepatoma cases had received cytotoxic therapy (with a variety of drugs) and a few had been treated with radiation therapy.

### METHODS

Six to 8 ml. of venous blood were collected by venepuncture (using all-glass syringes with disposable needles) and transferred immediately into stoppered glass tubes. After standing for $1\frac{1}{2}$ hours at 37° C. the tubes were centrifuged (3000 r.p.m. for 15 minutes) and 2 ml. of the supernatant serum pipetted into 125 ml. tall Pyrex beakers for digestion using a mixture of 5 ml. nitric acid and 2 ml. perchloric acid. The serum-acid mixture was covered and gently heated in a fume cupboard until all organic matter was destroyed leaving only a few drops of residual liquid. This residue was diluted to 10 ml. in a volumetric flask for subsequent zinc measurement. Zinc assay was performed on a Techtron AA4 atomic absorption spectrophotometer using a standard of pure zinc metal dissolved in nitric acid. Three separate aliquots of serum digest were measured, together with duplicate reagent blanks which were taken through the digestion procedure since even A.R. grade reagents contained detectable zinc contamination. All glassware used was decontaminated with hot aqua regia followed by thorough rinsing with distilled water. The instrument setting were as follows:

| | |
|---|---|
| Wavelength: 2139 Å. | Slit Width: 300 microns. |
| Air pressure: 15 psi. | Lamp Current: 6 mA. |
| Acetylene Flow: setting $\pm$ 4. | Burner: AB-41. |

Serum samples showing evidence of haemolysis after centrifugation were discarded. None of the blood samples were collected less than 1 hour postprandially.

### RESULTS

The serum zinc values found in the Bantu patients with malignant hepatoma and the controls are shown in Table I and Fig. 1. Statistical analysis of the values in the two groups indicate that the difference in the serum zinc values is not significant. No correlation was noted between serum zinc levels and any therapy administered to the cancer patients.

TABLE I.—*Serum Zinc Levels in the Controls and Hepatoma Patients*

| Group | No. cases | Range | Serum zinc ($\mu$g.%) Mean | S.D. |
|---|---|---|---|---|
| Controls | 40 | 59–183 | 100·5 | 29·2 |
| Hepatomas | 31 | 68–360 | 139·0 | 65·5 |

### DISCUSSION

Use of the atomic absorption spectrophotometer has greatly simplified accurate measurement of zinc in blood (Prasad, Oberleas and Halstead, 1966). By this technique normal plasma zinc levels are in the region of 100 $\mu$g.% (Prasad, Oberleas and Halstead, 1966; Davies, Musa and Dormandy, 1968). The results obtained in the present series indicate that the mean serum zinc level of healthy South African Bantu males closely approximates the plasma values reported for Caucasians in other countries.

We have been unable to substantiate the finding of elevated serum zinc concentrations in association with malignant hepatoma reported by Karlinsky and Roomere (1967). All but four of the serum zinc levels fall within the range of the control group. On a hypothetical basis there appears to be no reason to anticipate increased serum zinc levels in hepatocellular carcinoma, for while it is known that normal liver tissue contains considerable amounts of zinc (Olson, Heggen, Edwards and Gorham, 1954), Butt and Higginson (1957) have found mean hepatic zinc to be subnormal in Bantu cirrhotics. Local epidemiological studies have established that between 80 and 100% of hepatomas in the South African Bantu develop in such cirrhotic livers (Berman, 1951; Higginson, 1956; Becker and Chatgidakis, 1961). Low plasma zinc is a notable feature of alcoholic

FIG. 1.—Shows the individual serum zinc levels in the 40 controls and 31 hepatoma cases.

cirrhosis (Vallee, Wacker, Bartholomay and Robin, 1956; Vallee, Wacker, Bartholomay and Hoch, 1957). No definite explanation can be offered for the four elevated serum zinc levels encountered although the hepatomas in these four patients may have supervened in non-cirrhotic livers. Judged on the findings of Davies, Musa and Dormandy (1968) in patients with carcinoma of the bronchus, radiation or cytotoxic drug therapy do not result in elevated plasma zinc concentrations. The effects of such treatment in liver carcinoma cases, however, may not be comparable.

Although the mean serum zinc value (139 $\mu$g.%) in the 31 patients in the present series is somewhat higher than that of the controls (100·5 $\mu$g.%), the difference is not statistically significant. It therefore seems unlikely that the mean serum zinc value of 132 $\mu$g.% in the 12 cases reported by Karlinsky and Roomere (1967) represents a statistically significant increase.

As has recently been pointed out (*Lancet*, 1968) the status of blood and tissue zinc in relation to neoplastic disease in general is far from clear and merits further careful examination.

### SUMMARY

Serum zinc estimations have been performed by means of an atomic absorption spectrophotometer on 31 Bantu males suffering from primary liver cancer. A

mean serum zinc value of 139 μg.% was found as compared with a mean of 100·5 μg.% in a control group. This difference is not statistically significant. However, in four of the hepatoma cases elevated values (205 to 350 μg.%) were encountered.

We are grateful to the South African Primary Liver Cancer Research Group and to Dr. E. W. Geddes and Dr. N. de Moor for allowing us to study patients under their care; also to the photographic unit of the Department of Medicine for Fig. 1.

### REFERENCES

ADDINK, N. W. H. AND FRANK, L. J. P.—(1959) *Cancer, N.Y.*, **12**, 544.
AUERBACH, S.—(1965) *J. Lab. clin. Med.*, **65**, 628.
BECKER, B. J. P. AND CHATGIDAKIS, C. B.—(1961) *Acta. Un. int. Cancr.*, **17**, 650.
BERMAN, C.—(1951) ' Primary Carcinoma of the Liver '. London (H. K. Lewis), p. 100.
BUTT, E. M. and HIGGINSON, J.—(1957) *Acta Un. int. Cancr.*, **13**, 599.
DAVIES, I. J. T., MUSA, M. AND DORMANDY, T. L.—(1968) *J. clin. Path.*, **21**, 359.
HIGGINSON, J.—(1956) *Br. J. Cancer*, **10**, 609.
HIGGINSON, J. AND OETTLÉ, A. G.—(1957) *Acta Un. int. Cancr.*, **13**, 602.
KARLINSKY, V. M. and ROOMERE, P. A.—(1967) *Excerpta med. Cancer*, **15**, 384.
*Lancet*.—(1968) Editorial ii, 268.
OLSON, K. B., HEGGEN, G., EDWARDS, C. F. AND GORHAM, L. W.—(1954) *Science, N.Y.*, **119**, 772.
PRASAD, A. S., OBERLEAS, D. AND HALSTEAD, J. A.—(1966) ' Zinc Metabolism ' Illinois (Thomas), p. 27.
VALLEE, B. L., WACKER, W. E. C., BARTHOLOMAY, A. F. and HOCH, F. L.—(1957) *New Engl. J. Med.*, **257**, 1055.
VALLEE, B. L., WACKER, W. E. C., BARTHOLOMAY, A. F. AND ROBIN, E. D.—(1956) *New Engl. J. Med.*, **255**, 403.
VIKBLADH, I.—(1951) *Scand. J. clin. Lab. Invest.*, **3**, suppl. 2.
WOLFF, H. P.—(1956) *Klin. Wschr.*, **34**, 409.